# Practical Observations on Diseases of the Heart, Lungs, Stomach, Liver, &c. Occasioned by Spinal Irritation

**Published:** 1836-01-01

**Authors:** 


### PRACTICAL OBSERVATIONS ON DISEASES OF THE HEART, LUNGS, STOMACH, LIVER, &C., OCCASIONED BY SPINAL IRRITATION: AND ON THE NERVOUS SYSTEM IN GENERAL, AS A SOURCE OF ORGANIC DISEASE. Illustrated by Cases. By *John Marshall*, M.D. 8vo, pp. 172. London, Oct. 1835.

WE profess ourselves to be physiognomists in the title-pages and aspects of books, and somewhat inclined to the doctrine of *predestination*, as respects the names of authors. The subject of a work, too, often gives rise to a kind of *divination* as to the profit or loss that is likely to result from perusal of the volume. The name of the author—the appearance of the title-page—the size of the volume before us, made favourable impressions on the mind. The *first* assured us that a man of talent and experience had been occupied in the construction of the book—the *second*, or title-page, was short, expressive, yet simple. This looks well. The *third* (size) proved that the author was not a book maker by profession, as little profit could be expected from a six shilling volume—except in the way of *reputation*—the most honourable and legitimate source of emolument.

Dr. Marshall, as an Army Medical Officer, distinguished himself, towards the close of the war, by his zeal and talents, while our victorious troops occupied the capital of LA BELLE FRANCE, and for the last twenty years, has cultivated his profession in private practice. We are always glad to meet an old soldier or sailor, when competing for civil honours. There is an *honesty* about them that insures a favourable reception, and we seldom fail to find them rewarding us by bringing something useful into the field of science or literature.

Our readers will remember the extended analysis which we lately gave of the work of Messrs. Griffin on Spinal Diseases. It appears that Dr. Marshall has been engaged nearly eighteen years in the investigation of the same subject. When the work of Messrs. Griffin was published, his own was ready for the press; and he determined to print it before he consulted the work of Messrs. G. on the same subject. After it was printed, and before the preface was struck off, he very naturally ventured to peep into the volume in question, and while he was gratified by many remarkable coincidences, he was startled by some discrepancies. The latter, however, were easily reconciled by a little consideration of the different subjects and circumstances in the two cases.

It may be proper to state that, in the years 1815 and 16, our author had ample opportunities of prosecuting pathological researches in the British Military Hospitals in France. He then, like many others, thought that morbid anatomy was the polar star of medicine, and that it would guide him through all the labyrinths of our intricate science. He accordingly cultivated it with all the ardour of a young enthusiast. He was appointed to a medical charge in the Hospital of St. Louis, and "had unceasing opportunities of devoting himself to his favourite pursuit." On the cessation of war, he retired into private practice, with undiminished ardour in the cultivation of pathological anatomy.

"The more assiduously I pursued it, however, the more fully did I become aware of the truth, that morbid anatomy, however indispensable to the scientific practice of our profession, never could be to me, or any other physician, the *infallible* guide to diagnostics I had so fondly hoped to find it." 3.

Yes. This we have repeatedly urged in this Journal. Morbid structure is the consequence, rather than the cause of morbid function. At all events, *functional disorder* long precedes organic disease—and, therefore, *symptomatology* must be our first and most important study.

"Constantly did cases in private, as they formerly had in hospital practice, come under my observation, where, on *post-mortem* examination, no lesion or structural disease was found adequate to account for the symptoms during life: and, again, not a few presented themselves, where severe lesion and structural disease had actually existed, and no complaint had ever been made during life which could have led to the suspicion that such was the case." 3.

These and other observations led Dr. Marshall to the conclusion, that the NERVOUS SYSTEM was to be explored.

"It is an extraordinary, and, as far as I am aware, a hitherto almost unnoticed, law of the nervous system, that pain excited by severe pressure, or other morbid action at the root or origin of a nerve, or in any of the ganglions, is referred, not to the point where the cause exists, but to the distal extremity of the offended nerve." 4.

Dr. Marshall, in a note, discovers a passage in Sir Charles Bell's works, where this phenomenon is noticed. But, had his acquaintance with medical literature been more extended, he would have known that this fact has been almost universally acknowledged for a long time past. We need hardly remind our readers, that we have inculcated this doctrine for many years. The case of neuralgia which Dr. Marshall quotes from this Journal, in 1828, where the pain was referred to the fingers of the amputated hand, affords a familiar example. Dr. M. is inclined to give the nervous system the *initiatory*, in all or almost all morbid actions—and we have little doubt that he is near the truth. At the same time we must recollect, that the condition of the blood and other fluids influences the nervous system, and that the initiatory may thus be in the vascular apparatus, though unseen and unknown till another series of morbid actions comes to be set up. The following case is instructive.

"A gentleman, while skating, fell upon the ice, and received a contusion in the lumbar region, from which he did not at first perceive any great inconvenience. He shortly afterwards, however, began to find himself affected with alarming weakness of the lower extremities, and with retention of urine. The weakness rapidly increasing, almost to paralysis, the back was examined, and 'tenderness to touch being found present, the case was treated as a spinal one.' The remedial treatment is not condescended upon; but it is said that considerable amendment in the weakness of the lower extremities took place; in the functions of the bladder, however, no such amendment occurred. Incontinence supervened upon retention of urine, and in six weeks after the patient came under medical treatment, he expired.*

On a *post-mortem* examination, the whole viscera were found in a healthy state except the kidneys, which were 'gorged with very dark blood,' and several small abscesses formed in them. No morbid appearance could be detected in the spinal column; and 'the spinal marrow being found to all appearance perfectly healthy,' '*proves*,' says my Correspondent, 'that there could be no real morbid action of the spine nerves. Therefore I conceive that this forms a very curious example of *diseased kidney simulating spinal irritation*.'

From this diagnosis I beg leave completely and entirely to dissent. I conceive the *ratio symptomatum* in this case to be, that, at the period the patient fell upon the ice, the renal nerves received an injury or concussion so severe, as shortly afterwards to produce paralysis of them.

The bloodvessels of the kidneys, and those organs themselves, being thus deprived of nervous energy, became incapable of duly performing their functions. Hence the engorgement of 'dark blood,' and the breach of structure, found after death. The assertion that the spinal chord being found 'to all appearance healthy,' is an irrefragable proof that no real spinal irritation could have existed, is not borne out by fact." 12.

The following cases, which we shall abridge, go to an illustration of the same principle.

C. E. returned from Guyana to this country in 1818. He had resided 37 years abroad, where he spent much of his time in ornithological pursuits, in the woods of Guyana. About 15 years before leaving that country, he received several slugs in his body, while quelling an insurrection. Two of these, he supposed, remained unextracted, and to these he attributed his sufferings and bad health. One of these could be felt, though rather deeply seated, in the left lumbar region. The other had entered the left side, followed the course of the rib, and lodged near the spine, close to the 7th dorsal vertebra. Dr. M. thinks the patient was deceived on this point. His health never recovered after this event.

"He frequently complained of head-ache, nausea, and want of appetite; and he began to experience attacks, which gradually increased in frequency and severity, of numbness, cramp, and tremors of the lower extremities, especially of the left. Sometimes these assumed the character of violent spasm, attacking him so suddenly as more than once to occasion his falling to the ground. At the same time, the agony of pain darting down the limb was so great, that when attacked, as he frequently was, while seated in company, or walking with a friend, it was with the utmost difficulty he suppressed a scream. The only relief he had was to grasp the muscles of the thigh as tight as possible with both

---

* "As the mode of treatment is not condescended upon, we cannot judge whether it was judicious or not, and, indeed, it appears probable that disease in the kidneys had made considerable progress before medical advice was taken.

In such a case, I should have been inclined to exhibit leeches very freely over the whole lumbar region, and also frequent and long-continued frictions, with some stimulating liniment. At the same time, if the patient's constitution could stand it, I would have resorted to general depletion, with a view to relieve the system."

hands, and in a few minutes it passed away as if it had never been. At these times the voice became tremulous, and the speech faltering, and continued so for a longer period than the cause lasted : for the last five years of his life, this symptom became constant, and so severe as to make it difficult to understand what he said." 15.

Sometimes he was suddenly struck on the left side of the chest, as if by the fist of a strong man, or by a shock of electricity, occasioning the most acute anguish, and succeeded by a sense of burning. These attacks gradually increased in frequency and severity, till life became a burthen. In 1823, Dr. M. extracted the slug from the lumbar region. He experienced considerable relief. Dr. M. was solicited to try the removal of the other, but declined. Afterwards a surgeon in Birmingham made a rough and minute examination, coming to the conclusion that a foreign body existed in the part, and recommended an operation. Dr. M. had felt something like a foreign body himself, but conceived that it was not the slug. Strange to say, this substance, whatever it was, suddenly disappeared, and never afterwards could be found, either in the living or the dead body !

" Not very long after his return home (from Birmingham), he was seized with one of the spasms described, while in the act of mounting his horse, and was in consequence thrown upon his back with great violence. On recovering from the shock, he did not appear to have sustained any material injury; but from this time forward there was a rapid increase of suffering. The spasms of the limbs, and the sensation of receiving violent blows on the anterior part of the thorax, became much more frequent. The burning pain in the chest became permanent; and so severe, that the poor sufferer often awoke from sleep screaming for assistance, from the idea that he was struggling through a house on fire, or was from some cause enveloped in scorching flames. His sufferings were sometimes—not always—mitigated by the application of leeches, of anodyne, and cooling lotions, and the habitual use of the Black Drop; but no remedial measures employed gave more than a temporary relief." 17.

After this, he was distressed by carbuncles on the back—and, in two years from 1824, Dr. M. discovered a small pulsating tumour at the angle of the left scapula, too clearly indicating an aneurism of the thoracic aorta. Here follows a very curious phenomenon.

" On drawing a silk handkerchief along the left side of the thorax (over the spot where the burning sensation was complained of), from the dorsal to the sternal aspect, no unpleasant sensation whatever was produced ; but the moment this movement was reversed, by drawing the handkerchief from the sternal to the dorsal aspect, it elicited a scream of agony from the patient, and caused him to start from one side of the bed to the other." 18.

We need not pursue the melancholy details of this deplorable case. The tumour gradually increased, as did the sufferings of the patient—though many of the symptoms were such as could not well be traced to aortic aneurism. He exacted a solemn promise from Dr. M. that a most accurate examination, *post mortem*, should be made !! The following were the appearances, as traced by Mr. Buchanan, of Dumbarton.

" On examining the viscera of the abdomen, we found them all perfectly sound. The lungs were healthy, but the left was pressed almost flat between the pleura costalis and the aneurismal tumour, which nearly filled the left cavity of the thorax, occupying the arch, and the greater portion of the thoracic aorta.

On removing the tumour from the thorax, we found that the vertebral extremities of the ribs, from the 4th to the 7th, were entirely obliterated; as also were the sinistral portion of the bodies of the 5th, 6th, and 7th vertebræ, laying the theca vertebralis entirely bare along the whole body of the 6th vertebra; so that at this point the medulla spinalis must have been directly pressed upon by the tumour, and constantly exposed to its pulsations; yet it exhibited no morbid appearance whatever.

The tumour itself was chiefly composed of a light grayish substance, easily divisible into layers, which greatly resembled half-dressed leather in appearance and toughness. In the body of it we found several detached portions of the ribs, lying in irregular positions. The whole internal coat of the aorta was ossified, exhibiting an appearance as if covered with fish-scales. Considerable portions of it were so involved in the tumour, and so altered in structure, that but for this quantity of ossific matter, we should have been unable to trace it. The heart was enlarged, and pushed almost wholly under the sternum, and its walls thicker and firmer than usual; but no portion of ossific matter was discoverable in it, save at the tips of the tricuspid valves. The columnæ carniæ were remarkably firm.

Though examined with most particular attention, no morbid appearance could be detected, not even the slightest blush of inflammation, at the anterior part of the thorax, where the acute and agonizing sensation of burning pain had been experienced for so long a period during life." 20.

We may fairly infer that the above aneurismal disease was of very slow growth, and, therefore, we have little doubt that the neuralgic pains were its legitimate product throughout the whole course of the malady. We must detain our readers with some particulars of the second case. It occurred in a less exalted, but, perhaps, not less philosophic personage.

A negro, upwards of 80 years of age, applied to our author, after the death of the foregoing patient, presenting a striking coincidence of symptoms with those already described—and this led at once to the conclusion, that the cause was the same in both cases.

" He complained of severe burning pain on the left side of the linea alba, from the umbilicus to the ensiform cartilage; covering a very considerable portion of the left, but never at any time extending to the right side of the mezial plane; and also of the sensation of receiving sudden and violent blows upon the region of the stomach, where the sense of burning most prevailed. The same peculiar irritation was produced as in Mr. E.'s case, by drawing a silk handkerchief from the sternal to the left dorsal aspect.

At the time he first applied to me, I could not, on the most careful examination, detect any tumour externally; but in the course of the Summer, one which pulsated simultaneously with the heart and radial arteries, gradually developed itself in the upper part of the left hypochondriac region, immediately under the short ribs, and extending from the spine. It increased with amazing rapidity, and in a short time occupied the whole left side of the abdomen, reaching below the crest of the ilium. From this time till he expired, which was about six months afterwards, the agonies this wretched man endured were such as to baffle all description. Pains resembling those of the most acute rheumatism, but alternating with cramps and spasmodic twitchings, racked his limbs; the shrivelled and palsied looking extremities of which were at all times deadly cold. Severe head-ache was constantly present, which he vainly strove to relieve by a tight bandage round the brow.

I have already mentioned that Mr. E., from the most enlightened and benevolent motives, gave me his dying injunctions to examine his body; and it is remarkable that this poor uninstructed Negro did the same—'Ah, Massa, Massa!' he often repeated to me, ' whenever poor John dead, you open him's inside, and you see what dis terrible ting is, and you know how cure other body.'—About thirty hours after death had released him, I accordingly examined his body. On laying open the cavity of the abdomen, the viscera were found upon the whole healthy, but pushed over to the right side, and the aneurismal tumour occupying the left hypogastric, lumbar, and iliac regions. The tumour consisted of solid matter, of a buff colour, which could be separated into distinct layers, resembling leather. A small quantity of coagulated blood was found at the inferior or sacral aspect of the tumour; and in that part of it which had occupied the cavity of the ilium, detached pieces of the cartilaginous parts of the short ribs were found imbedded. As far as I was able to trace the aorta, it was ossified throughout. On prosecuting the examination into the thorax, the ravages were found to be very great. The pulmonary vessels contained a considerable quantity of dark blood. The heart was pushed completely under the sternum; was small, but in no other way remarkable. The left lung was pressed flat against the mediastinum, and anterior part of the chest, by the enlarged aorta, which occupied the whole left cavity of the thorax; and, in descending into the abdomen, had carried the short ribs along with it, dislocating them at their heads, where they join with the dorsal vertebræ; and by its perpetual pulsations, it had extensively absorbed the bodies of the dorsal vertebræ themselves; at one part, along the whole length of the seventh: the medulla spinalis was denuded even of the theca, and lay perfectly exposed; but, as far as we could ascertain, there was no breach of continuity in the medulla itself, nor did it appear otherwise than healthy." 22.

The enlightened, and the philosophic European, requested his mortal remains to be carefully examined—but, at the same time, left a solemn injunction that every particle of these remains should be deposited in the grave. Poor Sambo made no such stipulation! And accordingly a part of the spinal column, including the diseased vertebræ, and also a part of the tumor, were preserved, and are now in the possession of Mr. Morgan, of Glasgow. We agree with our author in thinking that, "if these cases are not considered corroborative of the theory he has ventured to advance, he has little hope from those which he now proceeds to lay before the reader."

The first section is on "diseases of the heart and large vessels simulated by nervous irritation." We shall pass entirely over the observations respecting the brilliant discoveries of Bell and Bellingeri. From the dates of various cases in this work, it appears that Dr. Marshall was engaged about the same time as Mr. Bell, in prosecuting the same inquiry. The text then of Dr. M.'s investigations is this:—*that morbid action at the root or ganglionic junctions of a nerve excites pain, disturbance in the circulation, and, ultimately, disease in the structures upon which the extremities of that nerve are ramified.*" This view of things naturally led our author, in all doubtful cases, to examine the spine as the seat of the principal nervous tissues, and to apply external remedies suitable to the case.

"So far from finding the spine, or spinal nerves, uniformly the seat of this, I have very frequently met with cases, as Case third, where a very high degree of organic disturbance existed, yet there was present little or no irritation of the spine; no complaint of pain there having ever been made, and manipulation causing no distress: nevertheless a careful and persevering application of local remedial treatment, terminating in a perfect cure, gave irrefragable evidence that the source of disease had been rightly apprehended; but that the sympathetic, par vagum, or phrenic nerves, and not the spinal, had been the primary seat of morbid action.

It has been most successfully demonstrated by Bichat and Sir Charles Bell, that the system of sympathetic nerves (as if in scorn of their name,) is incapable of conveying the sensation of pain. 'They may,' says Sir Charles, 'be cut or pinched in the living body without causing pain;' but it does not follow that they are therefore incapable of being irritated, debilitated, or diseased, so as to impair their energies, and render them a certain source of disease in the organs dependent upon their influence for health and vitality." 27.

We are now prepared to examine the cases on which our author's deductions depend. They are eight in number. We must abbreviate all those which we notice.

*Case* 1. (1821.) A young lady, aged 21 years, small stature and florid complexion, caught cold in her 18th year, and became threatened with phthisis. From this she recovered in the Summer; but had attacks of inflammation in the bowels, chest, and head in the Autumn. On the 28th October our author saw her, and considered her to be dying. Depletion had been carried to the utmost.

"I afterwards learned from a medical friend, that a few hours after I left her, a profuse, cold, clammy perspiration, the usual precursor of dissolution in such cases, came on, and in about half an hour she sunk into a state of insensibility; which all around her, save one individual, looked upon as death. The features wore all the peculiar characters of death—the breath and pulse were gone—not even the faintest throb of the heart could be felt—the whole surface of the body was cold and clammy; the jaw dropped, and the joints of the upper extremities stiffened—yet no argument would convince this individual that the cherished object of her fondest affection had indeed expired—and she persevered in using every means of resuscitation. For more than an hour these were wholly unsuccessful; but at length a very slight twitch in one of the fingers of the left hand was observed. Symptoms of re-animation gradually increased, and at the end of six hours, respiration and the motion of the heart were fully re-established; but the prostration of strength was so great that the eyes remained shut for several days. The power of speech was gone, and that of deglutition during the same period so imperfect, that only liquids in very small quantities, could be got over. The emaciation was so great, that more than one medical gentleman who saw her at this period pronounced, that from *that cause alone* it was impossible she could recover." 35.

She convalesced to some extent, when a new train of symptoms set in. When at rest, the pulse was 56 to 60; but very slight motion raised it to 100 or 140. Much agitation rendered the pulse innumerable, the patient complaining that the heart seemed to fill the whole thoracic cavity. The pulsations could be felt in all parts of the chest—face flushed—eyes prominent—sense of immediate suffocation. On the subsidence of these phenomena, great exhaustion remained. She could only lie in a half-recumbent posture during these attacks. Auscultation confirmed Dr. M. that organic disease of the heart existed. The father of the young lady, an old and talented physician, was inclined to think the complaint was occasioned by "peccant matter pressing upon the nerves of the heart and large blood-vessels." He therefore applied antimonial frictions to the spine. A plentiful crop of pustules ensued, and discharged much purulent matter. The urgent symptoms gradually subsided, and in the course of three weeks, entirely disappeared. She is now the mother of a family.

In such cases as the above, and we have seen several, the very *violence*

of the paroxysm has always led us to suspect nervous irritation rather than organic disease. But when rest and quietude of mind brought the action of the heart below par, we never afterwards entertained a doubt. We see young practitioners, every day, forming the most gloomy diagnoses and prognoses, on such occasions.

The case is, however, exceedingly curious and instructive from beginning to end. Those who despise such cases are no *clinical* practitioners. They are hankerers after doctrines, but despisers of the facts on which all sound medical theories must rest.

*Case* 2. (*July* 1821.) A young gentleman, aged 21 years, had enjoyed excellent health up to the present illness. He became clerk in a mercantile house in Liverpool, and soon began to complain of debility, dyspnœa, violent palpitation of the heart, loss of appetite, and emaciation. A physician advised him to return to his friends, as he was labouring under organic diseases of heart. When he arrived at Portglasgow—

" His appearance, at this time, was ghastly in the extreme. He was much emaciated ; the skin was sallow and shrivelled; the features sharp, and the expression of countenance anxious and disturbed; debility very great. He had severe pain of chest; frequent short dry cough ; no appetite; the bowels torpid; the urine of a most peculiar and fætid odour, depositing red sand on cooling. He was languid, and extremely disinclined to exertion of any kind, as it occasioned great increase of suffering; walking quick, ascending a stair, or any acclivity, occasioned such violent palpitation at the heart, and such a sense of instant suffocation, as compelled him to sit down every few minutes when engaged in any such exercise." 39.

Dr. M. was not quite satisfied with the diagnosis abovementioned. Percussion and auscultation detected no pulmonary disease; but the action of the heart was excessive. On examining the spine, he found about five inches of the dorsal portion very tender to the touch, and exhibiting a posterior curve. This let in a new light. Proper applications were made to the spine, and the digestive organs attended to. He was restored to perfect health, and has now continued 14 years in the enjoyment of that blessing.

*Case* 3. (*Oct.* 1830.) We cannot go into the particulars of this case, though it is very instructive. The subject of it was a boy, 12 years of age, who from being a fine youth, became reduced to the lowest ebb of emaciation and palpitation. He had been on the Valsalva treatment for organic disease of the heart. There was very little tenderness of spine. The treatment was changed. Light nutritious food was allowed, and the spine was rubbed twice a day with a stimulating liniment. A copious eruption came out on the back. He perfectly recovered under tonics and stimulants.

We would ask Dr. M. whether or not he suspected masturbation in any of these cases? We frequently meet with such symptoms resulting from this vice. A wrong treatment soon destroys their lives. We strongly suspect that such a state of things existed in the following case.

*Case* 4. (*Winter of* 1832.) James ——, aged 17 years, had enjoyed good health till the age of 15, when he was bound to the sea, and went two voyages to the West Indies. Soon after leaving port on the third voyage, he "*became affected with unaccountable weakness, particularly of the lower extremities, accompanied with difficulty of breathing, asthma, and palpitation of the heart on the slightest attempt at exertion.*" [These are the usual symptoms which we have observed in masturbation.] On his arrival at Greenock he was visited by two or three medical men, who considered the disease as hypertrophy of the heart. Some weeks after this decision, he was seen by Dr. Marshall. He was then in a deplorable condition. The chest was hollow—arms hanging to his sides—step hesitating—face sunk and emaciated—eyes languid and glazed—pupils dilated—face rendered swollen and purple on ascending the stairs—respiration laborious—pulse innumerable—appetite feeble—digestion disordered—urine turbid—motions of heart very violent. On examining the spine, a lateral curvature was detected, including the lower cervical and dorsal vertebræ. At this part, pressure occasioned a sob or sigh, with an uneasy sensation, and increased action of the heart. Leeches, succeeded by blisters, to each side of the spine—gentle aperients—alkaline bitters. In three weeks he was much improved, but far from being recovered. The progress of the case was slow, but progressive from this time; and, in twelve months the youth was restored to health. Four other cases, of similar tendency, are detailed; but we must pass them over, from want of space.

### DISEASE OF LUNGS OCCASIONED BY NERVOUS IRRITATION.

Believing that, in this and most other maladies, the first movements, of a morbid nature, are in the nervous system, Dr. M. endeavours to act upon that system, whether the disease be incipient or advanced. In the early stage, attention to the spine may do good—in the advanced stages, it can do no harm. There is no doubt that the accidental cures which St. John Long effected, and his successor, Ramage, now effects, were and are chiefly through the medium of frictions and counter-irritation. After some pertinent observations on the errors of the stethoscope, in inexperienced hands, and on the havoc produced among youth by the mania for over-education, Dr. M. proceeds to relate six cases of phthisis treated on the plan in question. We can only glance slightly at some of these.

The first case, though presenting formidable symptoms, was not, we think, phthisis at all. We suspect that it was more of hooping-cough than pulmonary disease. About the middle of December she was pronounced to be decidedly consumptive.

> "At this time she could scarcely walk across her room, and her appearance was quite phthisical. The pulse was generally from 100 to 120. The pain of chest was very severe, and the *paxoxysms of cough so violent that the unfortunate girl was wont to slide from her chair to the floor, when she felt them coming on, and there she lay nearlg convulsed.* No expectoration ever took place; but occasionally, when unusually severe, she vomited from half a pint to a pint of colourless water; and very often blood was brought up in small quantities." 64.

Frictions were ordered over the spine, and ultimately a crop of antimonial pustules were brought out on the column. The symptoms then amended rapidly, and she recovered her health. We can only notice one more case.

*Case* 2. "I found her extremely emaciated; the complexion of a peculiarly sallow hue; lips bloodless; eyes sunk, and yet staring; countenance expressive of great anxiety and suffering; complains of stitches in various parts of the chest, and of a fixed pain, covering a considerable extent in the centre of the sternum, reaching to the scrobiculis cordis. Frequent, hard, hacking cough, which greatly aggravates this pain and the stitches, and is occasionally accompanied with expectoration of mucus slightly tinged with blood. Great pain of head, sometimes most severe at the occiput, sometimes across the forehead, at other times diffused over the whole skull. Night perspirations are very severe, and debility so great, that when out of bed she snatches at each successive chair for support, as she moves through the room. The respiration is quick and hurried, greatly excited by motion; and the palpitation at the heart is then so violent 'as almost to take away the breath;' the shoulders are pulled up, and the chest hollowed; cannot make a deep inspiration; the voice peculiarly feeble; catamenia reported regular; bowels rather torpid; urine scanty, and peculiarly fætid; tongue flat, yellow, and slimy at the posterior portion; pulse 120, small, and very feeble; thirst considerable; appetite very poor and capricious." 66.

We agree with Dr. M. that few will deny the formidable character of the foregoing symptoms. Finding, however, that there were no actual proofs of organic disease of the lungs, Dr. M. felt inclined to view the malady, "as one of long-neglected nervous irritation," and therefore examined the spine. On drawing his fingers gently down along the column, no pain was experienced; but on increasing the pressure, no sooner did he reach the middle and lower portion of the vertebræ, than the patient winced, drew herself forward to escape from the fingers of the examinator, and complained of great pain in the sternum.

"On repeating the pressure still more firmly, she gasped for breath; the pain in the breast covered a larger portion; and the cough was severely excited; at the same time a most disagreeable thrilling sensation darted down the inner side of the thighs to the very soles of the feet. The curvature in the dorsal portion of the spine was very evident, even without a plummit." 67.

Eighteen leeches were ordered to the painful vertebræ—gentle friction over all the other parts of the spine, and also over the thorax. The bowels were regulated by laxatives—and tonics were also employed. The diet was to be generous, but not stimulating. The recumbent posture was enjoined.

"The leeches were applied at intervals of two and three days; and before a week had elapsed, the sternal pain began to give way; and, as it were, to *recede* from its first station. At each successive application of the leeches it seemed to retreat towards the spine, at the same time lessening in severity. If, trusting to these favourable appearances, the leeches were omitted for a day or two longer than usual, the pain advanced again towards the sternum, always retreating on a renewal of vigorous applications to the spine. The general health at the same time made a sensible advance, and all the urgent symptoms declined in severity. I then applied strips of blister, an inch broad, on each side of the spinous processes in the dorsal region, keeping them open as long as seemed prudent, then allowing them to heal, opened them again a little farther up or down the column, occasionally alternating them with leeches. The progress of the case under this mode of treatment was by no means *steadily* onward. It seemed at times to stand still; at others, almost to recede, instead of advancing. Nevertheless, when I considered that the hectic was subdued, the cough abated, and the flesh improving, I would not allow myself to despair. As the summer advanced, I sent her to the mildest and most inland situation that could be selected, for change of air; and, as in all cases of nervous irritation, this produced a most beneficial effect; she returned home in high spirits, greatly increased in flesh, and every thing in her case wearing a favourable appearance. By the end of July, every symptom of phthisis having completely disappeared, she was sent to the Island of Bute, where she enjoyed sea-bathing for several weeks, rapidly progressing to a state of perfect health, On her return home, it would have been difficult to recognize in the blooming, plump young woman she appeared, the same emaciated, and to all appearance dying person, I beheld her at my first visit in February." 68.

Although we think Dr. Marshall should have given us more authentic facts as to the physical signs in the above and in other cases, yet we are disposed to attach considerable importance to his cases, and to strenuously urge our brethren to attend to his admonitions.

### Asthma.

We shall quote the first case from this section, as one of the best.

" On the evening of 9th Jan. 1829, I received a hurried call to *A. H.*, the son of a respectable farmer, a stout lad of 14 years of age, accustomed to constant out-of-door work. The message bore that he had been suddenly seized with *croup*. On my arrival, I found him sitting at the fire-side quite composedly, and was informed that the attack had gone off as suddenly as it came on. The face, however, retained all the appearance of a recent and violent struggle, being swelled and bloated, with streaks of a livid hue. The eyes looked starting from their sockets, and the conjunctiva was suffused: the respiration hurried; pulse quick. From these appearances, joined to the description given of the attack by those who witnessed it, I was convinced it had not been one of croup, but of spasmodic asthma.

On requesting him to strip, I found the chest well formed and developed, and percussion excited no uneasiness whatever. On proceeding to examine the spine, my hand no sooner came in contact with the second, third, and fourth dorsal vertebræ, than it elicited a gasp from the patient, like that caused by a sudden plunge into cold water; and he complained that it seemed as if it would cause a second attack of all his previous sufferings and breathlessness. I could not, however, repress my curiosity to repeat the experiment, and did so several times, invariably with the same results. There was not the slightest displacement or twist of any of the vertebræ, nor did the same pressure that caused the gasping, cause any pain to the parts. The whole column was apparently healthy.

Satisfied that I had discovered the source of the spasmodic attack, I ordered two dozen of leeches to be applied over and around the spot were pressure occasioned the gasping: these to be applied every few days, and if the attacks of asthma did not cease to return, blisters to be applied, and kept up as an issue. The bowels to be kept open by mild purgatives, combined with tonics of iron and quinine.

Under this treatment the attacks of asthma declined in violence and frequency; and in two months the boy was restored to perfect health. He grew very rapidly during this time and immediately after; has, as far as I know, never experienced any recurrence of asthma; and became remarkably hale and robust." 77.

Three cases of simulated disease of liver are given by Dr. M. Excepting jaundice, however, there are no symptoms of diseased liver—or very few, in any of these cases, while there are many that would lead the judicious practitioner to suspect the spine. Take the following extract, for instance, from the first case.

" *J. R* ———, aged 29, cook in a gentleman's family in Greenook.—Has hitherto enjoyed good health. Consulted me in consequence of extreme debility, which renders her unable to move about or take the slightest exercise. If she walks a little distance, or ascends a stair, the fatigue is so intense as to oblige her to sit down every two or three minutes; her heart palpitates violently, the breathing becomes rapid and oppressed, and faintness frequently supervenes. She complains of a short dry cough, pain in the chest, from the sternum, extending round the sides; loathing of food, and constant acidity of stomach. Voice weak and husky; talking a few words completely exhausts it, so that she stops and breathes hard.." 79.

On examination, there was discovered an angular curvature of the spine, in the centre of the dorsal region. This led to an appropriate treatment and ultimate recovery.

Dyspepsia is a disorder which apes many others, and is itself imitated by spinal irritation. Four cases are related by Dr. Marshall—we shall make way for one of these.

" A young lady, ætat. 12, very tall of her age, and slender; dark eyes, hair, and complexion; complains of constant acidity of stomach, disinclination to food, amounting to nausea; acid eructations, accompanied with pyrosis; what comes up is occasionally so sour as to set the teeth on edge; constant languor, and aversion to exercise; frequent headache, and fits of the most unaccountable dejection and irritability. The bowels are torpid; the tongue foul; breath peculiarly fetid in the morning; pulse low and irregular; flesh and colour altered very much for the worse. One uncommon symptom is a constant craving for acids, so that it requires considerable attention to prevent the child drinking up vinegar, or sucking lemons, whenever she can by any means procure them.

Observing that this young lady's dress constantly hung off the left shoulder. I was induced to examine the state of the spine, and found it exhibiting a double curvature—the true Italic *f*, with a very well-marked gibbosity of the ribs on the right side, and great tenderness to touch in the cervical and dorsal vertebræ." 83.

The recumbent posture and appropriate remedies effected a recovery.

### Diabetes.

One case of this disease is given by Dr. Marshall. The patient was a farmer, who had enjoyed good health till his present illness. He had been over-excited in driving cattle over Highland mountains, in the Autumn of 1832, and was very much exposed to the weather, without other covering than a great coat. Shortly afterwards his strength began to fail, and his flesh to emaciate; yet his appetite was voracious. He had urgent thirst; and voided large quantities of limpid urine. When he consulted Dr. M. he was emaciated to a skeleton, and voiding from four to five gallons of urine in twelve hours. The urine was limpid and sweet. Thought himself a " dying man." Dr. M. had long entertained an opinion, that diabetes depended on " irritation or debility of the nerves supplying the stomach and urinary apparatus." He was, therefore, very much rejoiced to get hold of this case. He at first ordered some tinct. ferri muriat. and a laxative pill at night; but this plan availed nothing, and he was ordered to have a strong antimonial ointment rubbed along the spine. The eruption came out very tardily; but, at length, a copious crop was produced along the whole spine. "Shortly after their appearance, the symptoms began to subside with a rapidity far surpassing his most sanguine expectations." The quantity of urine gradually diminished, and became saline instead of saccharine. The appetite became natural, the thirst abated, and, in a few weeks, he was able to walk 14 miles in the day. He continued well up to the time Dr. M. left Scotland.

With this remarkable case, we must close a rather extended notice of this work. Numerous other cases are detailed, of tabes mesenterica, chorea, and anomalous affections which could not be classed under any head. We are very far from undervaluing (as some of our clever contemporaries do) this unassuming publication of Dr. Marshall. We are confident that it deserves serious attention, and that it will excite considerable interest in the minds of the practical portion of the profession, whatever the hypercritics may say or think to the contrary. Dr. Marshall, like most other authors, may have a HOBBY, and may be fond of riding the wee beastie rather farther than the wee beastie likes; but we are confident that the worthy Doctor travels generally in the right road, and very often reaches home in security after his long journies. We trust that we have given him a lift on the road, which will not be disadvantageous towards either the master or the hobby.

